# Total body irradiation practice patterns: insights from a national survey

**DOI:** 10.3389/fonc.2026.1960445

**Published:** 2026-10-09

**Authors:** Zaheeda Mulla, Ali Balbaid, Ghada Aldosary, Wsam Ghandourh, Ismail AlDahlawi, Mohamed Bayoumy, Mohannad Alhumaid, Fahad Alrowais, Hussain Almerdhemah, Hiba Taha, Abdullah AlMohamad, Wasil Jastaniah

**Affiliations:** 1 Department of Oncology, King Faisal Specialist Hospital and Research Centre, Jeddah, Saudi Arabia; 2 Radiation Oncology Section, Department of Oncology, King Abdullah Specialized Children's Hospital, Ministry of the National Guard-Health Affairs, Riyadh, Saudi Arabia; 3 King Abdullah International Medical Research Centre, Riyadh, Saudi Arabia; 4 Department of Health Administration and Medical Information, College of Health Science at Al-Leith, Umm Al-Qura University, Makkah, Saudi Arabia; 5 Department of Radiation Oncology, King Fahad Specialist Hospital-Dammam, Dammam, Saudi Arabia

**Keywords:** extended source-to-surface distance (SSD), hematopoietic stem cell transplantation, total body irradiation, treatment planning, volumetric-modulated arc therapy (VMAT)

## Abstract

**Purpose:**

Despite the expansion of hematopoietic stem cell transplantation (HSCT) services, contemporary Total Body Irradiation (TBI) practices remain insufficiently characterized in many healthcare settings. This study aimed to establish a national baseline for TBI practice to support benchmarking, standardization, and quality improvement.

**Materials and methods:**

A cross-sectional online survey was distributed between May and June 2026 to seven Saudi centers that provided TBI services. The questionnaire collected data on service capacity, patient populations, dose fractionation, treatment techniques and delivery, planning methods, and quality assurance. A census sampling approach was used, inviting Radiation Oncologists, Medical Physicists, and Therapists. Data were analyzed using descriptive statistics in Python (v3.11).

**Results:**

All seven centers responded. One center (C) submitted two responses covering conventional extended source-to-surface distance (SSD) and volumetric-modulated arc therapy (VMAT) practices. Commissioned between 1980 and 2020, the TBI programs manage an estimated 386 annual cases (range: 12–120). Fractionated TBI (10–12 Gy, six twice-daily fractions) predominated. The extended SSD opposed-beam technique remained in use at six centers, with the umbilicus as the most common prescription point. At two centers, extended-SSD TBI was used alongside Intensity-modulated radiation therapy (IMRT)-TBI (VMAT and Tomotherapy. IMRT-TBI-based centers reported volumetric target-coverage objectives of 90% and explicit organ-at-risk (OAR) dose planning constraints, particularly for the lungs and kidneys. Treatment planning for IMRT-TBI involved treatment planning systems (TPS), whereas extended-SSD techniques used varied planning approaches, including in-house spreadsheets, pre-calculated templates, and, in some cases, TPS-assisted planning. *In vivo* dosimetry was performed at all centers. The absorbed dose-rate at the prescription point ranged from <10 to 30 cGy/min for conventional techniques. Three centers planned their initial implementation of IMRT-TBI within three years. The ability to control OARs was the main driver, while linear accelerator availability and staff training were key barriers.

**Conclusions:**

Although conventional extended SSD TBI remains the most widely used technique, TBI practice is transitioning toward IMRT-based delivery. National practice guidelines based on existing international recommendations may help standardize care, address implementation barriers, and support the delivery of high-quality TBI services across treatment centers.

## Introduction

Total body irradiation (TBI), a specialized radiotherapy (RT) procedure, is a critical component of conditioning regimens for hematopoietic stem cell transplantation (HSCT) (1). Despite international guidelines advocating fractionated regimens and specifying dose and organ-at-risk (OAR) constraints to mitigate toxicity, substantial global variability persists in TBI delivery techniques, dose fractionation, shielding, and quality assurance (QA) protocols (2, 3).

Recent surveys have documented diverse institutional practices and highlighted an emerging technical shift from conventional lateral-opposed beam techniques to intensity-modulated radiation therapy (IMRT)-TBI (4–7). In this study, IMRT-TBI refers to TBI delivery using both volumetric modulated arc therapy (VMAT) and helical Tomotherapy-based treatment). The advantages of modern IMRT-based TBI techniques include improved sparing of several organs at risk, enhanced dose homogeneity, and more precise target dose delivery (8–11), however they remain underutilized due to resource availability, workflow challenges, limited clinical experience and lack of standardized protocols (7). Nonetheless, the technical and clinical advantages of VMAT-TBI, suggest that broader adoption is likely in the coming decade, making it essential for national surveys to track the pace and pattern of this transition.

Saudi Arabia’s expanding HSCT program lacks a systematic assessment of current TBI practice patterns, representing a significant knowledge gap and limiting opportunities for quality improvement, resource optimization, and adoption of modern techniques. International studies have demonstrated that national TBI practice surveys provide valuable insights into current practices, identify areas of consensus and variation, and establish benchmarks for quality improvement (4–7).

## Methods and materials

### Study design and population

A cross-sectional survey was conducted in May–June 2026, targeting all seven centers providing TBI services in Saudi Arabia, which were identified based on a recent study (12). Eligible participants included Radiation Oncologists, Medical Physicists, and Radiation Therapists, with a census sampling approach ensuring comprehensive multidisciplinary input.

### Survey description and data collection

The survey, adapted from Li et al., assessed institutional capabilities, patient demographics, clinical indications, dose fractionations, treatment techniques, planning methods, QA procedures, and barriers to advanced technique adoption (4). Multiple-choice questions replaced open-ended items to improve data uniformity. The adapted survey was reviewed by a team of expert professionals representing each multidisciplinary team including a radiation oncologist, a physicist and a radiation therapist, before distribution.

### Ethical considerations

The study was approved by the King Faisal Specialist Hospital and Research Centre Institutional Review Board (IRB#2261192), Saudi Arabia. Consent was obtained electronically from one designated respondent on behalf of each center prior to survey participation, and data confidentiality was maintained. No identifiers were used in this study as the survey was anonymous.

### Data analysis

The data were cleaned and descriptively analyzed using Python (v3.11).

## Results

### TBI services background and general practice

All seven centers responded (100%) with a single multidisciplinary consensus response completed jointly by a radiation oncologist, medical physicist, and radiation therapist. Center C, however, submitted separate data for conventional lateral extended SSD and VMAT-TBI techniques to reflect the expanded clinical use of their IMRT-TBI. TBI services were commissioned between 1980 and 2020 (**Figure 1**). The reported experience with the technique varied: three centers had used it for 0–5 years, two for 5–10 years, and two for 15–20 years. IMRT-TBI started in 2025 in three centers (C, E and G). All centers confirmed VMAT capability of their linear accelerators with routine use in external beam radiotherapy treatment and with interest in developing national TBI guidelines.

**Figure 1:**
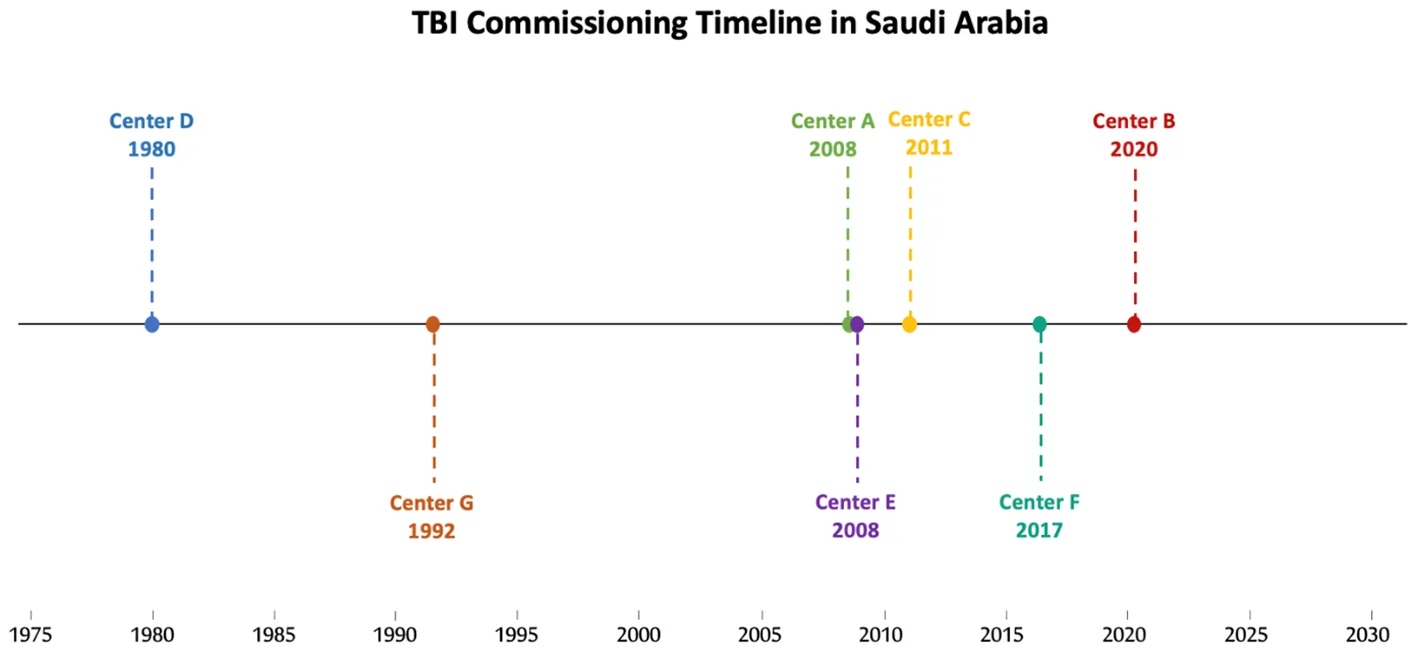
Timeline of TBI commissioning in Saudi Arabia.

### Patient volume

Typical annual caseload estimates ranged from 12 to 120 unique patients, totaling 386 nationally, treated with either conventional technique or IMRT-TBI. Adult low-dose TBI (151patients) was the largest category. Center F reported the highest overall volume (n=120) and Center D reported the highest high-dose caseload (n=60 adults) (**Figure 2**). The definition of pediatric age varied from 0–12 to 0–18 years, most commonly 0–14 years (four centers).

**Figure 2:**
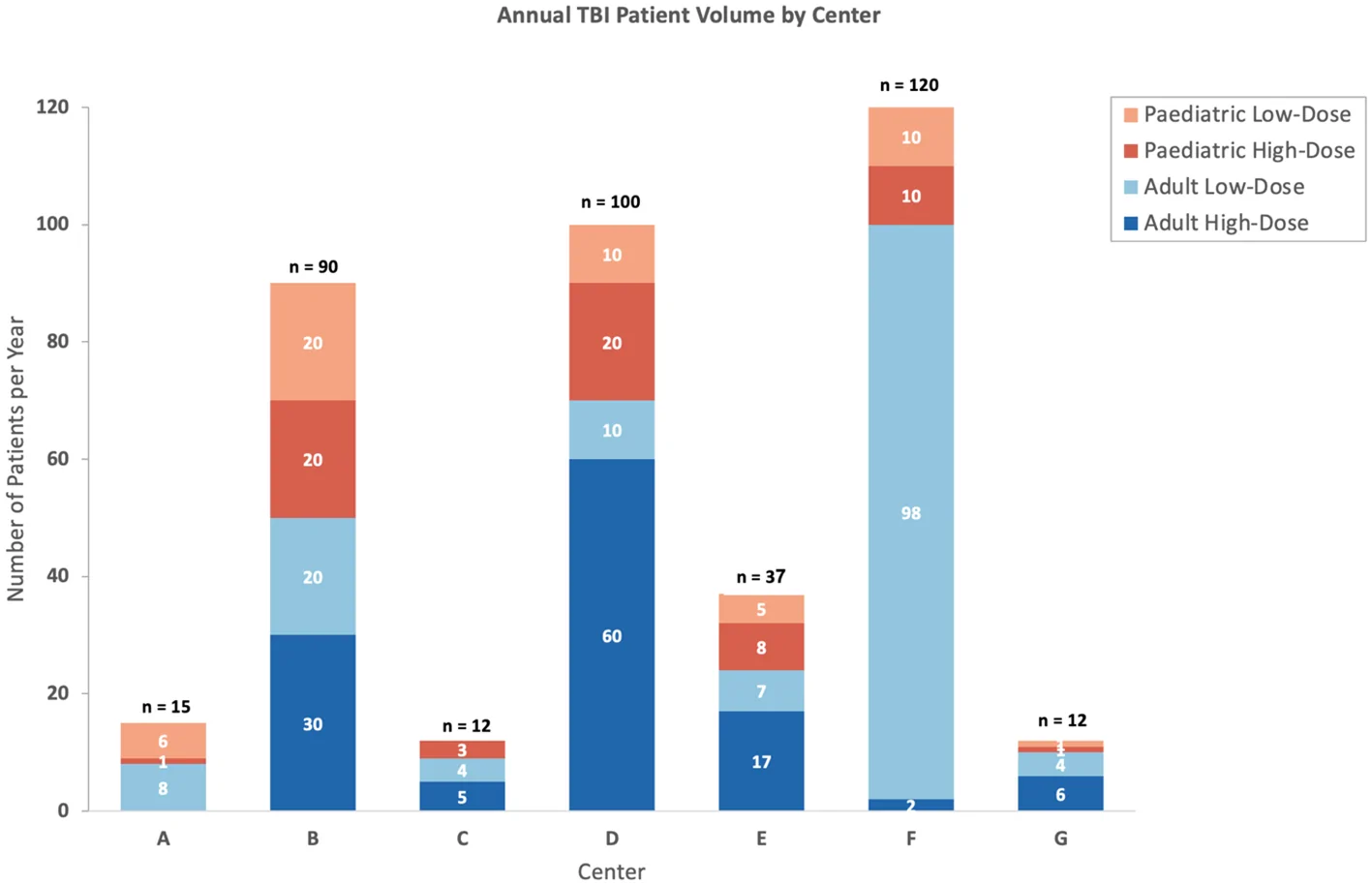
Annual TBI patient volume by centers. Pediatric age cut-offs vary by center: ≤14y (A, D, E, F), ≤12y **(B)**, ≤16y **(C)**, ≤18y **(G)**.

### Clinical indications, dose and fractionation

Acute lymphoblastic leukemia (ALL) and acute myeloid leukemia (AML) were universal indications. The other indications are listed in **Table 1**. High-dose TBI (10–12 Gy in six twice-daily fractions) was the most commonly reported regimen for adults and pediatrics. Single-fraction low-dose regimens were also used.

**Table 1:** Clinical Indications and dose prescriptions for TBI at each center.

	Clinical indications					Dose prescriptions	
Center	Acute leukemia (ALL, AML)	Sickle cell anemia & thalassemia	Myelodysplastic syndrome & multiple myeloma	Autoimmune diseases	Refractory lymphoma	Adult	Pediatric
A	✓	✓	✗	✓	✗	4Gy/1F 2-3Gy/1F	10-12Gy/6F BID 4Gy/1F 2-3Gy/1F
B	✓	✓	✓	✓	✗	10-12Gy/6F BID 2-3Gy/1F	10-12Gy/6F BID 2–3 Gy/1F
C	✓	✗	✗	✗	✓	10-12Gy/6F BID 4Gy/2F BID 2–3 Gy/1F	10-12Gy/6F BID
D	✓	✓	✓	✗	✗	10-12Gy/6F BID 4Gy/2F BID 4Gy/1F 2-3Gy/1F	10-12Gy/6F BID 4Gy/2F BID 4Gy/1F 2-3Gy/1F
E	✓	✓	✗	✗	✗	10-12Gy/6F BID	10-12Gy/6F BID
F	✓	✓	✓	✗	✗	10-12Gy/6F BID 4Gy/2F BID 4Gy/1F 2-3Gy/1F	10-12Gy/6F BID 4Gy/2F BID 4Gy/1F 2–3 Gy/1F
G	✓	✗	✗	✗	✗	10-12Gy/6F BID	10-12Gy/6F BID

BID, Twice daily dose; ALL, Acute lymphoblastic leukemia; AML, Acute myeloid leukemia.

For twice-daily fractionation, all centers used a minimum of 6 hours interfraction interval. The dose was most commonly prescribed to the umbilicus, either alone or in combination with another point for conventional TBI. For centers also using IMRT-TBI, a volume-based metric was utilized, covering 90% of the planned target volume (PTV)-body volume.

### Dosimetry, planning and quality assurance

CT simulation was used in IMRT-TBI and non-CT approaches were used predominantly in conventional TBI (**Table 2**). When CT was used, body contouring was performed by medical physicists (typically <15 minutes), while target volume and OAR contouring was done by physicists, radiation oncologists, or both, with times ranging from under 15 to over 90 minutes. The most commonly contoured OARs were the lungs and lenses, followed by kidneys, heart, testicles, and ovaries (**Table 2**). The dose constraint values reported in **Table 2** represent center-reported protocol parameters rather than treatment plan dosimetry derived from treated patients.

**Table 2:** Planning and dose constraints.

Center	CT-Sim used	Target volume	Planning aims %	OARs contoured	Lung dose constraint Gy	Kidney dose constraint Gy	Lens dose constraint Gy	Organs shielded	Shielding method	Planning method	Planning days
A	No	Body	+/-10	N/A	N/A	N/A	N/A	N/A	N/A	In-house spreadsheet	2-3
B	No	Body	+/-10	N/A	N/A	N/A	N/A	Lungs, Heart, Testicles	Lead Blocks	In-house spreadsheet	3-6
C ExtendedSSD C VMAT	No	Body	+/-10	N/A	N/A	N/A	N/A	N/A	N/A	Pre-calculated template	1-2
	Yes	Body minus lung (cropped 3mm from skin)	+/-15	Lungs Kidneys Lens	Mean Dose: <8	Mean dose: ≤10	Max Dose: <12	Lungs, Kidneys, Testicles, Lenses, Brain	MLCs	TPS	> 6
D	Yes	Body minus Lungs	+/-10	Lungs	Mean Dose: <8	N/A	N/A	Lungs Testicles	Lead Blocks	TPS, In-house spreadsheet	3-6
E ExtendedSSD E Tomotherapy	No	Body	+/-10	N/A	N/A	N/A	N/A	Lungs	Rubber lead	In-house spreadsheet	2-3
	Yes	Body minus lungs, kidney, brain, and liver (Tomotherapy)	+/-10	Lungs Kidneys Testicles, Ovaries, Heart, Lens	Mean Dose: <8	Mean Dose: ≤10	Max Dose: <12	Lungs Kidneys	MLCs	TPS, In-house spreadsheet	3-6
F	Yes	Body	+/-10	Lens Brain OAR structures for boost only	Mean Dose: <8	N/A	N/A	Lungs Testicles	Lead Blocks Testicular shield (Pb)	In-house spreadsheet	2-3
G	Yes	Body minus Lungs	+/-5	Lungs, Kidneys, Testicles, Ovaries, Heart, Lens	Mean Dose: <8	Mean Dose: <8	Max Dose: <8	Lungs	MLCs	TPS	1-2

VMAT, volumetric-modulated arc therapy; SSD, Source-to-Surface distance; N/A, Not applicable; MLCs, Multi-leaf collimators; TPS, Treatment planning system; CT-Sim, Computed tomography simulation; OARs, Organs at risk.

Treatment planning utilized treatment planning systems (TPS), in-house spreadsheets, or pre-calculated templates, taking 1 to over 6 days, and was performed exclusively by medical physicists. The reported planning objectives for dose homogeneity were ±10% (for all conventional techniques and Tomotherapy), ± 5% and ±15% for G and C, respectively, with VMAT-TBI. Refer to **Table 2** for reported lung, kidney and lens dose constraints. Independent Excel-based dose checks were performed for all patients across four centers.

*In vivo* dosimetry was performed at all centers, most of which used 3–8 detectors. Detectors were placed at multiple sites, most consistently the head, lung, umbilicus, and pelvis, with additional sites (neck, mediastinum, thighs, knees, ankles and under the arms) varying by center.

### Treatment delivery technique and setup

The most prevalent delivery technique reported was extended SSD using lateral opposed beams. Two centers employed VMAT. Center E used lateral beams, a moving couch technique, and Tomotherapy (for patient length < 135 cm). The other delivery techniques and energies used are listed in **Table 3**. The extended SSD ranged from 286 to 455 cm (212 cm for the moving couch), while VMAT and Tomotherapy centers treated at SAD of 100 and 85 cm, respectively. The supine position, was the most commonly used treatment position.

**Table 3:** Treatment delivery parameters.

Center	Delivery technique	Photon Beam energy MV	Patient position	Build-up materials	SSD or SAD setup	SAD/SSD distance cm	Pre-treatment imaging	Typical dose rate cGy/min	Dose rate Measured/Calculated
A	Lateral beams at extended SSD	15	Supine, Supine with knees bent	Rice Bags Perspex Beam Spoiler Bolus	SSD	286	None	<10	Calculated
B	AP beams at extended SSD	6	Lying lateral	Perspex Beam Spoiler	SSD	400	None	<10	Both
C	Lateral beams at extended SSD	18	Supine, Supine with knees bent	Rice Bags Perspex Beam Spoiler	SSD	455	None	10-20	Both
	VMAT	6	Supine, Supine with knees bent	N/A	SAD	100	Linac-based 2D/3D IGRT (i.e., EPID, kV, CBCT)	20-30	Calculated
D	AP beams at extended SSD Lateral beams at extended SSD	15	Supine, Lying lateral	Rice Bags Perspex Beam Spoiler Bolus	SSD	370	Portable X-rays (detector or unit)	10-20	Calculated
E	Lateral beams at extended SSD	6	Supine with knees bent, Supine	Rice Bags Perspex Beam Spoiler	SSD	400	Linac-based 2D IGRT (i.e., EPID)	<10	Calculated
	Moving couch technique	6	Supine with knees bent, Supine	Rice Bags Perspex Beam Spoiler	SSD	212	Linac-based 2D	20-30	Calculated
	Centralize Tomotherapy	6 FFF	Supine	N/A	SAD	85	MV-CBCT	<87	Calculated
F	Lateral beams at extended SSD	10	Supine	Rice Bags Perspex Beam Spoiler	SSD	450	None	<10	Calculated
G	Linac-based VMAT	6	Supine	Rice Bags	SAD	100	Linac-based 2D IGRT (i.e., EPID) Linac-based 3D IGRT (i.e., CBCT)	<10	Measured

SSD, Source-to-Surface distance; SAD, Source-to-Axis distance; MV, Megavoltage; MU, Monitor unit; Linac, Linear accelerator; CBCT, Cone-beam computed tomography; VMAT, Volumetric-modulated arc therapy; AP, Anterior-posterior; IGRT, Image-guided radiation therapy; EPID, Electronic portal imaging device.

Lung shielding was the most widely applied OAR protection reported; In two extended-SSD approaches, no physical lung shielding was used; instead, lung dose was modified through arm positioning and bolus application. Testicular shielding was reported for three approaches, kidney shielding for two, and heart, lens, and brain shielding for one approach each, with the latter reported for VMAT (**Table 2**). The most common method utilized for shielding was lead blocks, followed by multileaf collimator (MLC) shielding (VMAT technique) and lead rubber. Patient positioning included custom or in-house TBI beds, stretchers with custom mattresses, linac couch tops for VMAT, and aids such as head supports, Vacloc bags, and masks.

The reported absorbed dose rates at the prescription point for conventional and VMAT-TBI ranged from <10 cGy/min to 30 cGy/min, with a dose rate of <87 cGy/min reported for Tomotherapy at Center E. The dose rate values reported by the centers were determined by calculation, both measurement and calculation, or measurement alone (**Table 3**).

In contrast, the reported linac dose-rate settings ranged from 100 to 400 MU/min for conventional techniques, while Center C reported a reduced setting of 200 MU/min for chest arcs and 600 MU/min for other body regions during VMAT-TBI. Adult delivery times varied, with the longest reported for VMAT at Center C and generally shorter delivery times reported for pediatric patients.,

Pre-treatment imaging included portable X-rays for conventional TBI and IGRT for IMRT-TBI. (**Table 3**).

### Self-reported practice ratings

Ten aspects of practice were self-rated from 1 (poor) to 5 (excellent). Linac reliability and intra-departmental workflow scored the highest (mean 4.5 each), followed by dose delivery accuracy and *in vivo* dosimetry (4.2 each). OAR dose control showed the greatest variation (1–5, mean 3.5), reflecting the contrast between conventional centers with limited shielding and VMAT techniques with full optimization. The availability of backup-technique also varied (3–5, mean 3.9). Patient experience and dose uniformity accuracy each averaged 4.1, with scores as low as 3. (**Figure 3**).

**Figure 3:**
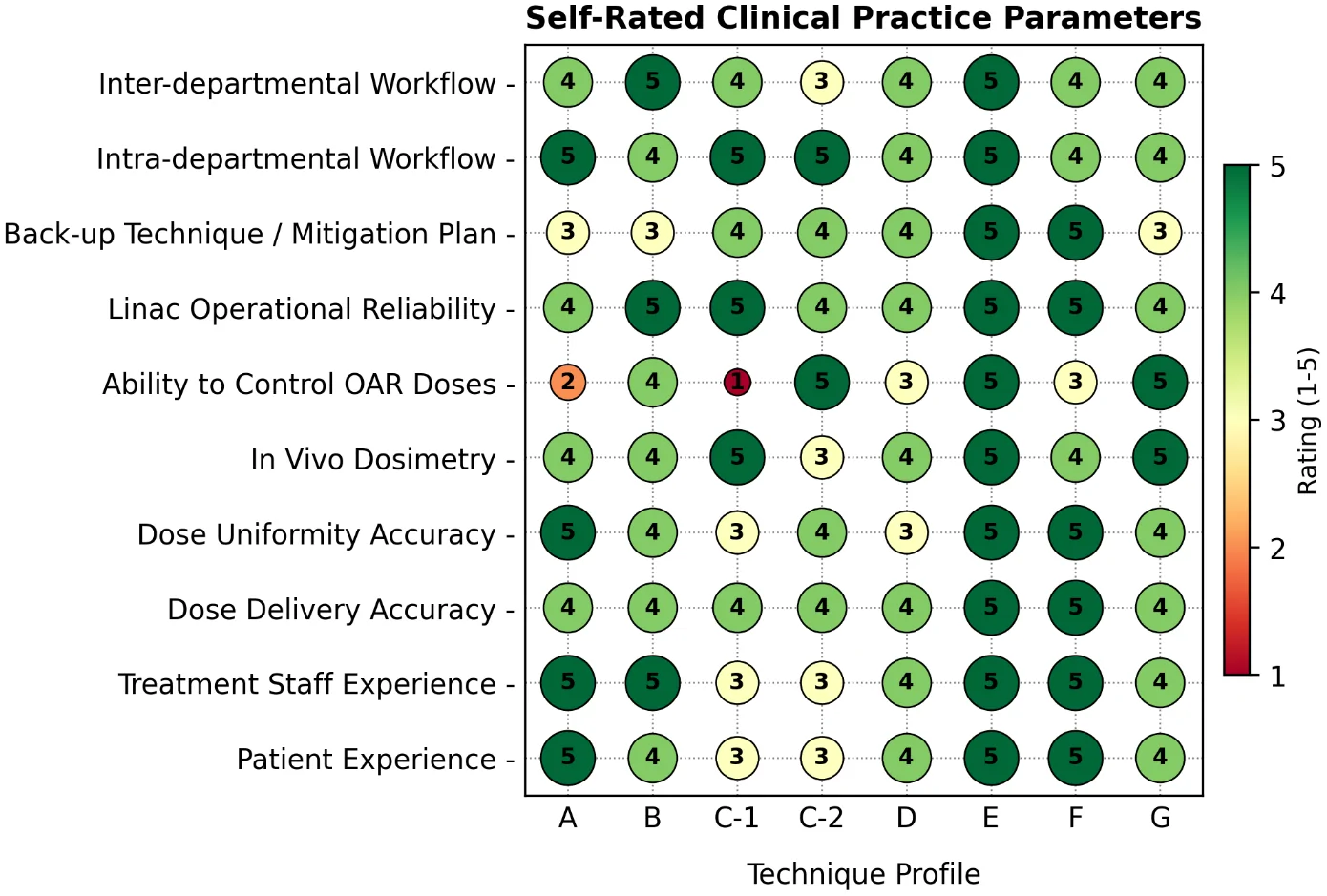
Self-rated clinical practice parameters by technique profile. Seven institutions contributed data, with Center C providing separate responses for its conventional extended-SSD technique (C1) and VMAT-TBI technique (C2), resulting in eight technique-specific profiles. A, B, C1, D, and F represented conventional extended-SSD practice; C2, E and G represented IMRT-TBI.

### Future readiness

Three of the seven centers planned their first implementation of IMRT-TBI within three years, except Center F. Center C was already using VMAT as its increasingly established default technique, while Centers E and G were using IMRT-TBI for selected cases. The most cited improvement priority was OAR dose control, followed by patient experience and prescription dose uniformity (**Figure 4**). The main barriers to VMAT adoption were linac slot availability and training (three centers each), interdepartmental workflow (two centers), and staffing and patient volume constraints (two centers each). (**Figure 5**).

**Figure 4:**
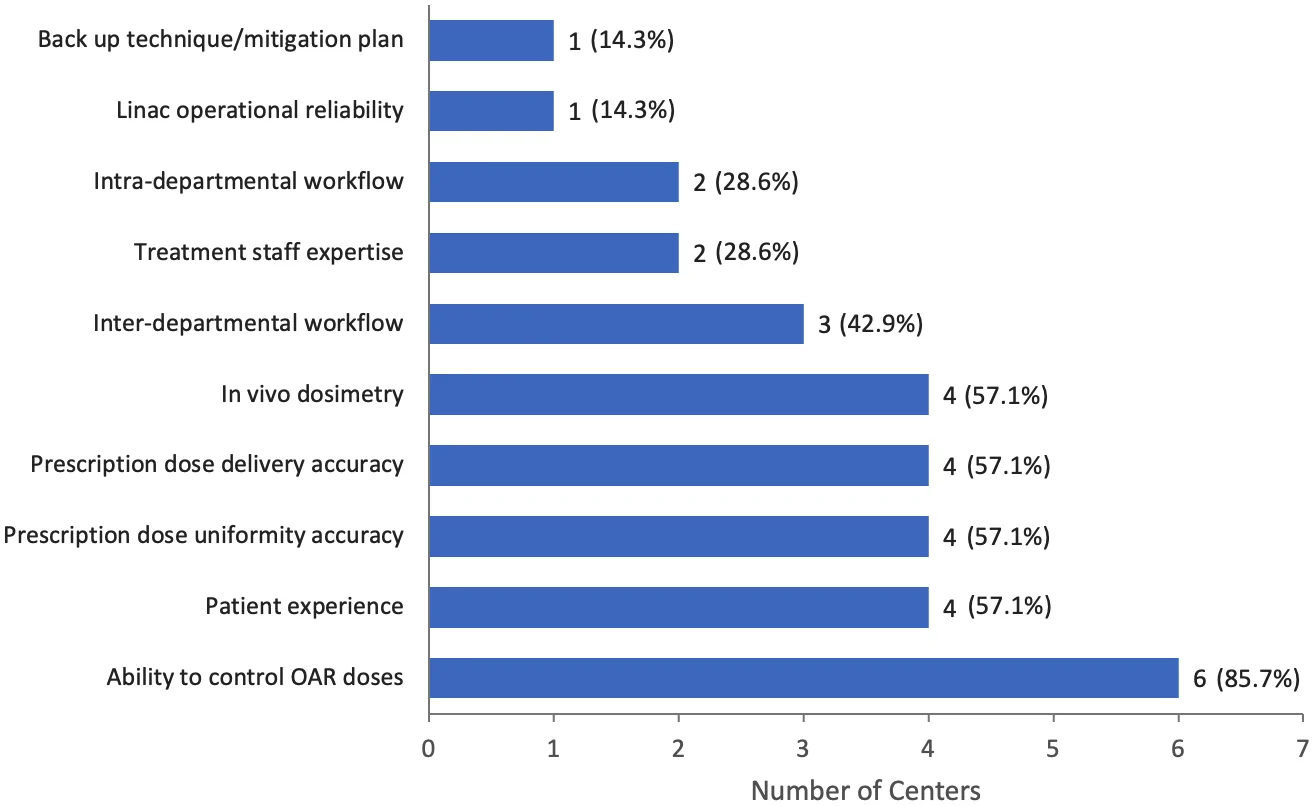
Aspects being actively improved.

**Figure 5:**
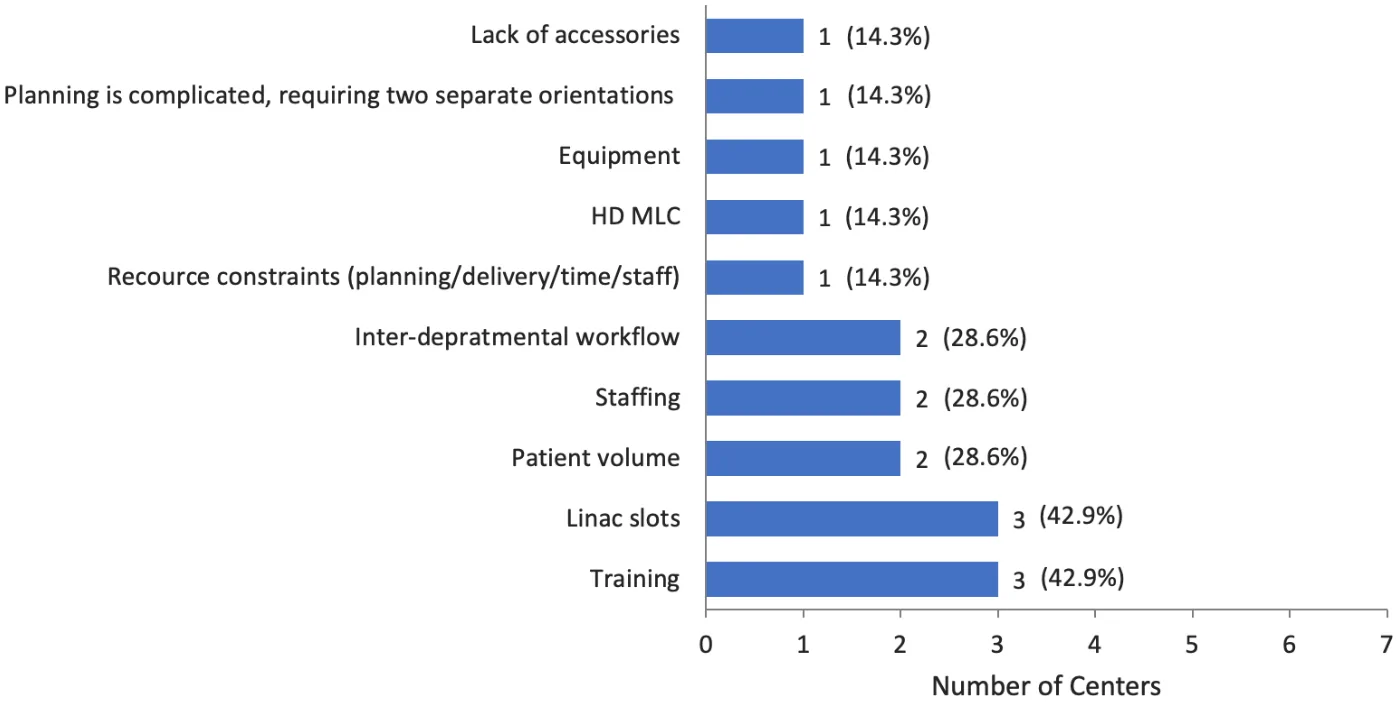
Perceived barriers to VMAT adoption for TBI.

## Discussion

Our survey provides the first national overview of TBI in Saudi Arabia. While several commonalities were identified, the participating centers provided a valuable opportunity to examine variations and evolving trends in TBI practice across Saudi Arabia.

### Patient volume and service growth

The combined annual TBI caseload across the participating centers highlights the established role of TBI within the national radiotherapy landscape. Variability in institutional experience is consistent with international practice, where TBI services are often delivered in specialized transplant centers (4). The data demonstrated that adult low-dose TBI was the most common treatment category, followed by adult high-dose, pediatric high-dose, and pediatric low-dose TBI, reflecting the diverse clinical indications for TBI.

The establishment timeline of TBI services (spanning from 1980 to 2020) illustrates progressive national capacity building, with three centers reporting the commissioning of IMRT-TBI in 2025. While pediatric age definitions have historically varied, the Saudi Health Council recently mandated a standardized definition for individuals under 18 years of age (13).

### Clinical indications and dose fractionation

Patients with ALL and AML were treated across all Saudi centers, consistent with the well-established role of TBI as a conditioning regimen for HSCT in patients with hematologic malignancies. The breadth of indications, included hemoglobinopathies and autoimmune diseases, reflecting both the diversity of transplant protocols and regional disease epidemiology, particularly the high prevalence of hemoglobinopathies in Saudi Arabia and the surrounding region (14, 15).

High-dose fractionated TBI (10–12 Gy in six fractions delivered twice daily) adult regimen was used by all but one center. However, all centers applied this regimen to pediatric patients. The Canadian survey reported near-identical fractionation dominance, with 12 Gy in 6 fractions used by 11 of the 12 TBI-performing centers. Notably, half of the Canadian centers also reported using a dose prescription unique to their facility, reflecting a similar degree of institutional variation to that observed in Saudi Arabia (**Table 1**; **Figure 3**) (5).

The six-hour minimum inter-fraction interval reported by all centers is consistent with radiobiological principles, allowing adequate repair of sublethal damage in normal tissues between fractions (2, 16). Low-dose single-fraction TBI (2–4 Gy) was used by most centers for adults and pediatrics, typically for a reduced-intensity conditioning regimen. This fractionation heterogeneity is consistent with international practices, where regimen selection is dictated by the transplant protocol, disease type, patient age, and comorbidities (3, 17). Unlike malignant indications, which commonly employ fractionated TBI to a total dose of 12 Gy, conditioning regimens for thalassemia and aplastic anemia frequently incorporate low dose TBI (18).

The diversity in dose prescription points and volume-based PTV-Body D90% reflects the transition from conventional point-based dosimetry to modern volumetric IMRT planning, where dose prescription to a volumetric metric enables a more rigorous evaluation of target coverage and homogeneity compared to traditional point-dose specifications (1).

### Technique variation and the transition to VMAT

The dominance of conventional extended SSD lateral beam techniques reflects the historically established clinical practice, while the emerging VMAT adoption signals modernization aligned with global trends (2). A systematic comparison with current international guidelines contextualizes this finding. The ACR–ARS practice parameter for the performance of TBI addresses both conventional and evolving volumetric modulated TBI approaches, reflecting the continued role of conventional techniques alongside the development of conformal delivery methods (1). The International Lymphoma Radiation Oncology Group [ILROG] consensus guidelines support the use of CT-based volumetric planning and conformal techniques for TBI, with the aim of improving dose homogeneity and facilitating organ-at-risk sparing (2). The European Society for Radiotherapy and Oncology (ESTRO) ACROP and European Society for Paediatric Oncology (SIOPE) recommendations, particularly for pediatric patients, similarly support highly conformal techniques such as IMRT-TBI to improve dose homogeneity, reduce radiation exposure to organs at risk, and thereby mitigate potential late effects in developing organs (3).

Among the three centers utilizing IMRT-based TBI, Center C reported an expanded adoption of VMAT-TBI in routine clinical practice whereas Centers E and G are using IMRT-TBI for selected patients and are currently in a transition phase. The Saudi baseline stands in contrast to the 2024 ANZ survey in which seven of 19 centers had already adopted modulated techniques (VMAT or helical Tomotherapy), while 12 continued to use large open fields (4). Regionally, the broader regional landscape mirrors this shift toward modernization; the King Hamad University Hospital Oncology Center in Bahrain and the Sultan Qaboos Comprehensive Cancer Care and Research Center (SQCCCRC) in Oman have introduced VMAT-TBI, while the Kuwait Cancer Control Center utilizes extended SSD modulated TBI (19).

Lung shielding is the most commonly used organ protection measure. Notably, the shielding of the lungs, kidneys, testicles, lenses and brain in Center C with VMAT application reflects the organ-sparing capabilities of VMAT-based TBI techniques (20).

Dosimetric studies comparing VMAT, helical Tomotherapy, and conventional techniques have demonstrated that conformal approaches reduce the mean lung dose by approximately 25% compared to 2D AP/PA techniques, with VMAT and Tomotherapy achieving lung mean doses in the 6–8 Gy range versus higher values with conventional lateral beams (10, 21, 22). VMAT has also been shown to increase PTV D90% by 6–7% compared with 2D techniques and provide additional sparing of the kidneys, gonads, brain, and thyroid (10, 21). However, with a lack of prospective data comparing conventional vs IMRT-TBI, it is not clear whether dosimetric advantages will translate into better outcomes.

### Dosimetry, planning, and quality assurance

CT simulation was used for all IMRT-TBI techniques, while conventional techniques primarily used non-CT-based approaches. International guidance increasingly recognizes CT-based three-dimensional planning as an approach that can facilitate assessment of dose distribution, dose heterogeneity, and organ-at-risk exposure, particularly with conformal techniques. The ILROG guidelines describe the increasing use of CT-based 3-D planning and conformal techniques, while also recognizing conventional approaches based on point measurements (2). The most commonly contoured OARs were the lungs and lenses, followed by the kidneys, heart, testicles, ovaries. This pattern aligns with international consensus recommendations, which emphasize the lung mean dose as the primary constraint for high-dose TBI, with additional consideration of the kidneys, lenses, and gonads (1, 23). Even when active sparing is not performed, contouring enables dose documentation, protocol compliance, and future evaluation of dose–toxicity relationships.

Treatment planning utilized TPS, in-house spreadsheets, or templates, with planning times varying from 1 to over 6 days, (for VMAT-TBI in Center C), reflecting the substantial time investment required for conformal TBI planning. Published institutional experiences report contouring times of several hours and optimization/dose calculation times spanning many hours to days for VMAT-TBI, with QA procedures adding several additional hours per patient (24–26). This workflow burden represents a major barrier to the broader adoption of VMAT (4).

According to the American College of Radiology (ACR) and American Society for Radiation Oncology (ASTRO) guidelines, dose inhomogeneity should generally be maintained within ±10% (1, 27), to ensure adequate target coverage while limiting excessive hot and cold spots. However, in VMAT-TBI, greater dose inhomogeneities may be acceptable than with conventional TBI to balance target coverage with organ-at-risk sparing (1). The ILROG similarly endorses ±10% homogeneity for conventional TBI but acknowledges that planned heterogeneity may be intentionally introduced in conformal techniques to achieve OAR sparing (2). The ESTRO/SIOPE recommendations permit selective dose reduction to OARs provided that adequate target coverage is maintained (3). Dose homogeneity of ±10% was the predominant planning objective among surveyed Saudi centers.

While the reported QA practices demonstrated strengths in the universal adoption of *in vivo* dosimetry to assess the dose delivered to the patient, independent dose verification, involving an additional check of the calculated dose using a separate calculation method or system, was performed in only half the centers, highlighting a key area for improvement. ILROG guidelines recommend multi-layered QA encompassing patient-specific pre-treatment verification and independent secondary dose calculation (2), and recent VMAT-TBI and Tomotherapy institutional series consistently implement portal dosimetry or diode/array measurements together with secondary calculation checks as standard (24, 25, 28, 29).

### Organ-at-risk dose control and clinical implications

Self-reported practice ratings revealed that OAR dose control showed the greatest variation. The ACR-ARS practice parameter highlights the evaluation of the mean lung dose as an important factor related to pulmonary toxicity for high-dose TBI regimens (1), and clinical reports emphasize lung sparing as a primary driver for adopting conformal techniques (21–23).

Early clinical series of linac-based VMAT and Tomotherapy TBI reported acceptable acute and subacute toxicity, with one cohort demonstrating reduced severe mucositis after implementing oropharyngeal mucosa sparing (30). The clinical value of the organ sparing is most clearly established for the lungs, where reductions in mean lung dose have been associated with lower rates of radiation pneumonitis (2, 31). Consequently, a mean lung dose constraint of approximately 8 Gy has been widely adopted in contemporary TBI protocols and is readily achievable with modulated TBI techniques (1, 3). The centers employing IMRT-TBI techniques reported planning objectives of a mean lung of <8 Gy and mean renal dose of <8–10 Gy and maximum lens of <8–12 Gy for adults and pediatrics, consistent with ACR–ARS, ILROG, and ESTRO/SIOPE thresholds (1–3).

The centers that identified OAR dose control as an area for active improvement indicated the perceived limitations of OAR sparing provided by conventional techniques. This finding, combined with the fact that many centers plan to adopt IMRT-TBI within three years, suggests that Saudi TBI practice is set to transform in the near future. However, replacing extended SSD with VMAT-TBI increases resource demand. A key barrier is that limited couch travel restricts the maximum treatable length in the superior–inferior direction, requiring patient repositioning between head-first (HF) and feet-first (FF) supine orientations in centers without rotational tabletops, increasing planning and treatment delivery owing to multiple isocenters and extensive image guidance (10). Furthermore, the use of multiple abutting fields complicates field matching, often introducing dose uncertainties at the junction interfaces. Additionally, the shift away from simultaneous whole-body irradiation has prompted concerns regarding the potential under-dosage of circulating leukemia cells due to the temporal gap in delivery (2).

### Self-rated practice and quality metrics

Dose delivery accuracy and *in vivo* dosimetry received consistently high self-rated scores, indicating a high perceived confidence level in dosimetric verification practices. Patient experience and dose uniformity accuracy also received high mean ratings. This was supported by qualitative feedback from Center B, which described extended SSD as “a reliable setup that ensures a positive experience for patients and staff, while maintaining dose delivery and accuracy.” However, both measures received low ratings from Center C, indicating variability in perceived performance across the centers. These findings represent the respondents’ perceptions of practice and should not be interpreted as evidence of objectively measured dose accuracy or uniformity.

The availability of backup techniques showed considerable variation, highlighting potential vulnerabilities in service resilience. Given the time-sensitive nature of transplant conditioning (2, 32), maintaining continuity of TBI services during equipment failure or unplanned downtime is essential (20). The development of contingency pathways, including backup treatment protocols (1) and inter-institutional referral agreements, may represent an important area for future national coordination.

### Future readiness and barriers to VMAT adoption

VMAT adoption within three years was the most commonly cited target. Center G has begun VMAT-TBI for patients <150 cm and plans to commission a rotating tabletop for taller patients in the future. Of the centers not planning near-term changes, Center C had already implemented and expanded their VMAT-TBI program, and Center F indicated that adoption of a new technique was not planned, stating that “our extended SSD technique is generally robust and reliable”.

The perceived barriers most commonly reported in VMAT adoption; linac slot availability, training, interdepartmental workflow challenges, staffing, and patient volume constraints are consistent with international reports (4, 22, 24–26, 33) and represent systemic challenges in radiotherapy. A national survey confirms this, with nearly 60% of departments reporting limited technical skills training, underscoring the critical need for workforce development and workflow integration (12).

Proposed solutions include auto-segmentation, automated planning, standardized recommendations, QA frameworks, and multicenter efforts to align Dose-Volume Histogram and OAR constraint practices (20, 23, 24, 34).

### The need for national guidelines

All Saudi centers supported the development of national TBI guidelines, reflecting the considerable variation in techniques, dosimetry, planning objectives, OAR constraints, QA practices, and dose prescription points observed across centers. Comparable international variations have similarly driven calls for standardization (5, 33). Similar international variation has prompted standardization efforts and published recommendations (3, 20, 23, 35). A national multidisciplinary TBI steering committee could adapt these guidelines to local practice while supporting workforce planning, benchmarking, quality improvement and equitable access to advanced TBI techniques.

### Limitations and future directions

Reliance on self-reported data and the mixture of institution-level and technique-level observations represent important limitations of this study. Potential differences in the interpretation of questions, together with the inclusion of centers undergoing practice transition at the time of the survey, represent important considerations when interpreting the findings. Other caveats include the lack of independent verification of reported data, variation in pediatric age definitions and the absence of patient-level dosimetric or clinical outcome data. Nevertheless, these findings provide a national baseline for prospective multicenter studies and support the establishment of a national TBI registry for longitudinal quality monitoring, research, and international collaboration.

## Conclusion

This survey of centers employing TBI practices in Saudi Arabia revealed a service landscape characterized by substantial patient volume, diverse clinical indications, and significant technical heterogeneity. The dominance of conventional lateral beam techniques at extended SSD, combined with widespread plans to adopt VMAT within three years at most centers, positions Saudi Arabia’s TBI practice at a critical inflection point. Building on existing international recommendations, a nationally coordinated approach could support the standardization of practice, to support the delivery of high-quality TBI services across treatment centers for patients undergoing HSCT.

## Data Availability

The raw data supporting the conclusions of this article will be made available by the authors, without undue reservation.
