# Supplementary material for: Total body irradiation practice patterns: insights from a national survey
Source: Front Oncol. 2026 Oct 9;16:1960445. doi: 10.3389/fonc.2026.1960445 (PMC13690712; doi:10.3389/fonc.2026.1960445)
Supplement: Supplementary file 1 [file DataSheet1.pdf]

Supplementary Appendix S1

Total Body Irradiation Practice Patterns: Insights from a National Survey

Do you consent to participate?

☐ Yes (Skip to Section 1)

☐ No

Section I. General Information

1. Name of centre – note all centres will be de-identified in publication, and referred to as ‘a’, ‘b’ etc

☐ King Faisal Specialist Hospital & Research Centre, Riyadh

☐ King Faisal Specialist Hospital & Research Centre, Jeddah

☐ Prince Sultan Military Medical City, Riyadh

☐ National Guard Hospital, Riyadh

☐ National Guard Hospital, Jeddah

☐ King Fahad Medical City in Riyadh

☐ King Fahd Specialist Hospital, Dammam

2. This survey was completed by: (check all that apply)

☐ Radiation Oncologist

☐ Medical Physicist

☐ Radiation Therapist

3. a. When was TBI first commissioned in your centre? ____________

b. How long has your centre’s current TBI technique (or similar) been operational?

If incremental changes have been made but are still fundamentally using the same technique then please answer as though it is the same technique.

☐ 0–5 years

☐ 5–10 years

☐ 10–15 years

☐ 15–20 years

☐ More than 20 years

4. How would you rate the following aspects in your current clinical practice?

(5 = Very Positive, 4 =Positive , 3 = Neutral, 2 = Negative, 1 = Very Negative)

a. Patient experience
b. Treatment staff experience
c. Prescription dose delivery accuracy
d. Prescription dose uniformity accuracy
e. In vivo dosimetry
f. Ability to control OAR doses
g. Linac operational reliability
h. Back up technique/ mitigation plan
i. Intra-departmental workflow
j. Inter-departmental workflow

5. Is there anything about your department's current TBI technique that your clinic is actively working to improve? (check all that apply)

a. Patient experience
b. Treatment staff expertise
c. Prescription dose delivery accuracy
d. Prescription dose uniformity accuracy
e. In vivo dosimetry
f. Ability to control OAR doses
g. Linac operational reliability
h. Back up technique/ mitigation plan
i. Intra-departmental workflow
j. Inter-departmental workflow

k. No

6. a. Is your clinic working to adopt a new TBI treatment technique in the next 3 years?

☐ Yes

☐ No

b. If so, which technique is your clinic planning to adopt?

☐ Anterior–posterior beams at extended SSD

☐ Lateral beams at extended SSD

☐ Moving couch technique

☐ Linac-based VMAT

☐ Tomotherapy

☐ Other, describe: ____________

7. Are there other aspects about your TBI practice (current or future) that you'd like to comment on?

____

Section II. TBI Practice

8. As per your departmental policy, how are paediatric patients defined (if applicable)?

☐ 0-14 years

☐ 0-18 years

☐ Other, Explain ___________

9. Who does the TBI Planning? (Check all that apply)

☐ Medical Physicists

☐ Dosimetrists

☐ Radiation Therapist

Patient Demographics

10. Approximately how many TBI patients do you treat per year? (specify for each, if none write "0")

10a. Total Number: ______
10b. Number of Adult high dose TBI e.g., 10-12 Gy (patients receiving ≥ 6 Gy over the course of treatment) ______
10c. Number of Adult low dose TBI e.g., 2-4 Gy (patients receiving < 6 Gy over the course of treatment.) ______
10d. Number of Pediatric high dose TBI e.g., 10-12 Gy (patients receiving ≥ 6 Gy over the course of treatment) ______
10e. Number of Pediatric low dose TBI e.g., 2-4 Gy (patients receiving < 6 Gy over the course of treatment) ______

11. In your department, which clinical indications are treated with TBI? (Check all that apply)

a. Acute leukemias (ALL, AML)

b. Myelodysplastic syndrome and multiple myeloma

c. Sickle cell anemia and thalassemia

d. Autoimmune diseases

e. Others, disclose: ____________

Dose Prescription

12. What are the prescribed dose/fraction regimens used in your department (Gy/Fx/Days) for adult cases? (check all that apply)

a. Adult: 10-14.4 Gy/6 fx BID
b. Adult: 4 Gy/2 fx BID

c. Adult: 4 Gy/1 fx
d. Adult: 2-3 Gy/1 fx
e. Other adult regimen (describe in Gy/ Fx/ Days): ______

13. What are the prescribed dose/fraction regimens used in your department (Gy/Fx/Days) for paediatric cases? (Check all that apply)

a. Paediatric: 10-14.4 Gy/6 fx BID
b. Paediatric: 4 Gy/2 fx BID
c. Paediatric: 4 Gy/1 fx
d. Paediatric: 2-3 Gy/1 fx
e. Other paediatric regimen (describe in Gy/ Fx/ Days): ______

14. For BID fractions, what is the minimum time between fractions? (Check all that apply)

☐ 6 hours

☐ 8 hours

☐ Other, describe: ___________

15. Where is the dose prescribed to? (check all that apply)

☐ Pelvis midpoint

☐ Umbilicus

☐ Lung midplane

☐ Shoulder midplane

☐ Other, Explain___________

16. Who prescribes the dose? (check all that apply)

☐ Haematology

☐ Radiation Oncology

☐ Both

Treatment Technique and Equipment

17. What delivery technique does your department employ? (check all that apply)

☐ Anterior–posterior beams at extended SSD

☐ Lateral beams at extended SSD

☐ Moving couch technique

☐ Linac-based VMAT

☐ Tomotherapy

☐ Other, Explain____________

18. What beam energy is used? (check all that apply)

☐ 6 MV

☐ 10 MV

☐ 15 MV

☐ 18 MV

☐ Other, describe: __________

19. What is the patient position? (check all that apply)

☐ standing

☐ sitting

☐ lying supine

☐ lying supine with knees bent

☐ lying lateral

☐Other, describe: __________

20. Does your department use an SSD or an SAD setup? (check all that apply)

☐ SSD

☐ SAD

21. What is the SAD/ SSD/where is the patient in relation to the Linac? (check all that apply)

☐ 100 cm

☐ 200 cm

☐ 250 cm

☐ 300 cm

☐ 350 cm

☐ 400 cm

☐ Other, describe__________

22. Which build-up material/scatter/spoilers etc. are used? (check all that apply)

☐ Rice Bags

☐ Perspex Beam Spoiler

☐ Bolus

☐ None

☐ Other, describe____________

23. Which organs are shielded during TBI? (check all that apply)

☐ Lungs

☐ Kidneys

☐ Testicles

☐ Ovaries

☐ Heart

☐ Lenses

☐ None

☐ Other, Explain____________

24. How is shielding achieved?

☐ MLCs

☐ lead blocks

☐ Not applicable

☐ Other

25. Which dedicated patient treatment positioning equipment are used? (check all that apply)

☐ Commercial TBI bed

☐ Custom/ in-house TBI bed

☐ Patient stretcher with custom mattress

☐ Rotating TBI couch top

☐ Standard linac couch top

☐ patient seat

☐ patient standing support system

☐ head support

☐ vaclok

☐ mask

☐ Other, Explain____________

26. How many working days are TBI treatments allocated for the treatment planning and approval steps (note that this does not include subsequent patient specific quality assurance, if applicable)?

☐ 1-2 days

☐ 2-3 days

☐ 3-6 days

☐ > 6 days

Treatment Delivery

27. a. What is the typical dose rate at the patient/prescription point? *e.g.* 6–8 cGy/min. (check all that apply)

    a. < 10 cGy/min

    b. 10-20 cGy/min

    c. 20-30 cGy/min

    d. > 30 cGy/min

    e. Other in cGy/min, describe:

    b. Please state whether this is measured / calculated

☐ Measured

☐ Calculated

☐ Both

28. What is the typical linac dose rate setting used? (check all that apply)

☐ 100 MU/min

☐ 200 MU/min

☐ 300 MU/ min

☐ 400 MU/ min

☐ Other, describe____________

29. Is in-vivo dosimetry carried out?

☐ Yes

☐ No

a. If yes, which type? (check all that apply)

☐ Ionization chamber

☐ Diodes

☐ TLD / OSLD

☐ Film

☐ Not applicable

☐ Other, describe: ____________

b. How many detectors are typically used?

☐ 0
☐ 1-3
☐ 3-5
☐ 5-8
☐ > 8

c. Describe where the detectors are typically placed: _________

d. How are the results utilized? (check all that apply)
☐ In-vivo dosimetry is not performed
☐ Reporting only
☐ Adjusting dose delivery
☐ Both

30. Is pre-treatment imaging used? (check all that apply)

☐ Yes, portable X-rays (detector or unit)

☐ Yes, linac-based 2D IGRT (i.e., EPID)

☐ Yes, linac-based 3D IGRT (i.e., CBCT)

☐ None

31. How long does it take to deliver a typical TBI treatment fraction (check all that apply)

a. Adult TBI:
☐ <30 min ☐ 30–60 min ☐ 60–90 min ☐ >90 min

c. Paediatric TBI:
☐ <30 min ☐ 30–60 min ☐ 60–90 min ☐ >90 min

Treatment Planning

32. How is treatment planning performed?

☐ Treatment planning system

☐ In-house spreadsheet

☐ Other, Describe: ___________

33. Is CT Simulation used for TBI planning?

☐ Yes

☐ No

If yes please Answer the questions below:

34. a. Who does the body contouring structure? (check all that apply)

☐ Medical physicist

☐ Dosimetrist

☐ Radiation oncologist

☐ Not applicable

☐ Other, Explain____________

b. Who does the target volume contouring? Note: If the entire body is the target volume, then state who finalizes it. (check all that apply)

☐ Medical physicist

☐ Dosimetrist

☐ Radiation oncologist

☐ Not applicable

☐ Other, Explain____________

c. Who does the OAR and shielding contouring? (check all that apply)

☐ Medical physicist

☐ Dosimetrist

☐ Radiation oncologist

☐ Not applicable

☐ Other, Explain____________

35. How long does it typically take to contour the:

Body: ☐< 0.25 hrs ☐0.25-0.5 hrs ☐ 0.5-1 hrs ☐ 1.0-1.5 hrs ☐ >1.5 hrs ☐ Not applicable

Target volume: ☐< 0.25 hrs ☐0.25-0.5 hrs ☐ 0.5-1 hrs ☐ 1.0-1.5 hrs ☐ >1.5 hrs ☐ Not applicable

OARs and shielding: ☐< 0.25 hrs ☐0.25-0.5 hrs ☐ 0.5-1 hrs ☐ 1.0-1.5 hrs ☐ >1.5 hrs ☐ Not applicable

36. Which structures (organs at risk) are routinely contoured?

☐ Lungs

☐ Kidneys

☐ Testicles

☐ Ovaries

☐ Heart

☐ Lenses

☐ Not applicable

☐ Other, describe:_____

37. What is your treatment target volume?

☐ Body

☐ Body -Lungs

☐ Body with margin to external contour

☐ Other, Explain___________

38. Please share your planning aims to the target volume.

☐ +/-5%

☐ +/-10%

☐ +/-15%

☐ Other, Explain___________

39. For high-dose TBI, which OARs are included in your planning considerations and what are the typical dose constraints used?

☐ Lungs : Mean Dose: ☐ < 8 Gy ☐ Other, state in Gy_____ ☐ Not applicable

☐ Kidneys : Mean Dose: ☐ ≤10 Gy ☐ Other, state in Gy_____ ☐ Not applicable

☐ Lenses : Maximum Dose : ☐ < 12 Gy ☐ Other, state in Gy_____ ☐ Not applicable

☐ Other OAR, name and state the associated dose constraint as a maximum, mean, or volumetric and provide the dose in Gy___________

40. a. Is software or spreadsheet based independent dose check performed?

☐ Yes, for all TBI patients

☐ Yes, occasionally (e.g., complicated TBI cases etc.)

☐ No

b. If yes, please describe ___________

41. a. Is detector-based patient specific QA performed?

☐ Yes, for all TBI patients

☐ Yes, occasionally (e.g., complicated TBI cases, as part of routine TBI quality assurance etc.)

☐ No

b. If yes, please describe ___________

Section III. Future Readiness

42. VMAT capability: ☐ Yes ☐ No

43. Barriers: ☐ Equipment ☐ Linac slots ☐ Training ☐ Staffing ☐ Patient Volume ☐ Interdepartmental workflow ☐ Other, describe: _____

44. Interest in national guidelines: ☐ Yes ☐ No

45. Interest in collaboration: ☐ Yes ☐ No
